# Navigating Medical School with Hearing Loss

**DOI:** 10.31729/jnma.8313

**Published:** 2023-10-31

**Authors:** Sangit Chhantyal

**Affiliations:** 1 Maharajgunj Medical Campus, Maharajgunj, Kathmandu, Nepal

**Keywords:** *communication*, *hearing loss*, *medical students*, *patients*, *speech*

## Abstract

Hearing impairment is a significant challenge faced by medical students and physicians, impacting their communication abilities and stethoscope use. This condition, though prevalent, remains underaddressed in medical education, with many institutions lacking explicit support systems and some even imposing exclusionary standards. This personal narrative chronicles the journey of a medical student who experienced a gradual and severe bilateral sensorineural hearing loss. This article also highlights the pivotal role of cochlear implants in significantly enhancing the author's academic and social life. Medical educators are encouraged to understand the challenges faced by hearing-impaired students and adapt their teaching methods to support them effectively.

## INTRODUCTION

Hearing is an important sense for medical students and physicians, making communication and stethoscope use possible.^1^ Hearing loss is currently the most common physical and sensory disability encountered in medical school. Many schools do not explicitly support accommodations, and some reject certain accommodations or have more exacting and exclusionary technical standards.^2^ Deafness is not a visible disability, although the hearing aids are conspicuous. Most physicians and medical students with hearing impairment want people to understand the obstacles they have to hearing, while simultaneously wanting to appear "normal."^3^ They become quite isolated, repeatedly failing at social engagement and finding it very difficult to understand what anyone is saying, particularly in locations with background noise.^4^ However, there is a lack of literature on the difficulties faced by hearing-impaired medical students and how medical educators can support them with their training.^5^

## MY EXPERIENCE

Bilateral sudden sensorineural hearing loss was diagnosed when I was 14 years old. However, at that time, it was mild to moderate in severity. Later, my hearing capacity gradually decreased over time until I could no longer recognize words despite perceiving sounds to a satisfactory level. Further investigation showed bilateral auditory neuropathy spectrum disorder (ANSD), a hearing condition where the ear receives sounds normally, but the signals to the brain get scrambled, making it difficult for a person to understand speech or other sounds clearly.^6^ While facing shock, grief and fear at the same time, the phase of denial made the situation even more challenging.^7^ The feeling of isolation and frustration made me gradually lose the closeness with my family, friends, and relatives. However, over time I gradually accepted my condition and was resilient, determined and hopeful that some technology may surely help in future.

By the time I entered my first year in medical school, I was nearly deaf bilaterally. With the fear of being noticed as a hearing-impaired student, understanding lectures were the most challenging and even minor noises were irritating. While having a hard time listening to people in groups, I preferred studying alone. As a result, my speaking frequency decreased which affected my speech in comparison to others and was painful in itself. My hearing loss made my learning very slow and difficult, but I made the decision to never give up.

During my early clinical year, my friends would write everything the professor and patients would say. I completely relied on them and it was tiresome and stressful. Besides, COVID-19 made everything difficult with everyone wearing face masks as recognizing facial expressions and lip reading was impossible. During the lockdown phase, I relied on auto-captioning of Microsoft Teams applications as Zoom had no captioning feature. My experience on Zoom was limited to reading slides and taking screenshots at that time as a general public.^8^

During my first year, I was donated a pair of hearing aids. I was excited about listening to everything, having conversations, sharing knowledge and having to laugh together with friends, family and relatives. I was hard working and also thought I would enjoy learning medicine. That did not happen. My hearing aids just amplified sounds but speech recognition was very poor. So, the first 3 years of my medical school were the same with lots of challenges on the way. Still, the amplification of sounds proved to be a great help for me.

At the end of my third clinical year, I had right-sided cochlear implant surgery. I did a lot of practice with an implant device. Although its performance depends on whatever social situation I am in, it brought significant improvement in my academics as well as social life. I could hear lectures, phone calls and group conversations. Although background noises were still a problem during conversations with patients as well as professors in the ward, I would just request them to take their masks out and speak louder so that it could be audible that way. I thrived my remaining medical school years successfully and until now.

I avoided approaching patients during the beginning of my clinical years for fear of being judged. Repeating was stressful for them like listening was for me. As a result, practical exams were challenging to tackle. In addition, the background noises inwards made it almost impossible to have conversations with patients.

After my cochlear implant surgery, my hearing was relatively better. My confidence to approach patients increased by introducing myself and my difficulties first. It became quite easier that way by gradually increasing my visits with different patients to ask about their conditions and do relevant clinical examinations properly. As a result, once painful practical exams became easier during my fourth and final year as I used a Thinklabs digital stethoscope for auscultation. I could hear all the heart, lung and bowel sounds. In this way, I learned how to have a good doctor-patient relationship despite being hearing impaired.

## WAY FORWARD

The number of hearing-impaired students and doctors within medicine will likely increase in the future. This type of problem necessitates the inclusion of targeted preventive medicine modules in medical curricula regarding modern technology and its health effects. As this type of hearing impairment once established, is irreversible but completely preventable and medical educators need to learn and adapt to these student's needs. Medical educators must become familiar with the challenges faced by hearing-impaired students and be aware of what they can do to enhance their learning experience and support them to achieve to the best of their ability. Furthermore, hearing-impaired students should disclose their disability, and ask people to make reasonable adjustments to help them hear.
